# Perioperative Outcome of Dyssomnia Patients on Chronic Methylphenidate Use

**DOI:** 10.1177/2324709614521398

**Published:** 2014-01-29

**Authors:** Nicoleta Stoicea, Thomas Ellis, Kenneth Moran, Wiebke Ackermann, Thomas Wilson, Eduardo Quevedo, Sergio Bergese

**Affiliations:** 1 Department of Anesthesiology, Wexner Medical Center, Ohio State University, Columbus, OH; 2 Department of Neurological Surgery, Wexner Medical Center, Ohio State University, Columbus, OH; 3 Department of Orthopaedics, Wexner Medical Center, Ohio State University, Columbus, OH; 4 Ohio State University, College of Medicine

**Keywords:** methylphenidate, general anesthesia, narcolepsy, hypersomnia

## Abstract

Methylphenidate is frequently prescribed for attention deficit hyperactivity disorder, narcolepsy, and other sleep disorders requiring psychostimulants. Our report is based on 2 different clinical experiences of patients with chronic methylphenidate use, undergoing general anesthesia. These cases contrast different strategies of taking versus withholding the drug treatment on the day of surgery. From the standpoint of anesthetic management and patient safety, the concerns for perioperative methylphenidate use are mainly related to cardiovascular stability and possible counteraction of sedatives and anesthetics.

## Introduction

Methylphenidate is one of the most prescribed psychostimulants. The drug is approved for treatment of attention deficit hyperactivity disorder (ADHD), the prevalence of adults with ADHD is estimated at around 4.4% (1), postural orthostatic tachycardia syndrome, and dyssomnia disorders—narcolepsy and hypersomnia as an off-label medication. Based on an overview published by Do It Now Fundation, in 2005, 1.9 million U.S. prescriptions were written for methylphenidate.

Methylphenidate has a pharmacological profile similar to all the sympathomimetics of the phenylethylamine class. General anesthesia in animal studies demonstrated interaction between amphetamines and halothane resulting in altered minimum alveolar concentration (MAC) requirements as well as intraoperative hemodynamic changes.^2^ Two case reports published in 1979, implicated amphetamines as the cause of serious intraoperative complications, leading to hesitancy to continue chronic stimulant therapy (for conditions such as ADHD, hypersomnia, narcolepsy, depression) during surgery.^3,4^

## Case Presentation

### Case 1

A 44-year-old female patient presented for surgical removal of a vocal cord cyst. The patient’s medical history was significant for fibromyalgia, depression, anxiety, gastroesophageal reflux disease, morbid obesity, degenerative disc disease, obstructive sleep apnea on continuous positive airway pressure, chronic asthmatic bronchitis, chronic hypertension, irritable bowel syndrome, hypokalemia, narcolepsy, and dysphonia due to vocal cord cyst, all of which were considered active problems at the time of surgery. Her hypertension had been adequately controlled with carvedilol and spironolactone. Preoperative pulmonary assessments and electrocardiogram interpretation were within normal limits. Preoperative laboratory data showed normal findings.

The patient had one year history of narcolepsy and cataplexy for which she was prescribed oral methylphenidate. Her dosage of methylphenidate was adjusted to extended release 10 mg PO, QID. At the time of admission, other medications consisted of albuterol, clonazepam, bupropion, cyclobenzaprine, hydrocodone-acetominophen, linactlotide, ondansetron, topiramate, zolpidem, hyoscyamine, fluticasone, docusate, dicyclomine, promethazine, rosuvastatin, tizanidine, tramadol, lisinopril, aspirin, and esomeprazole.

On the morning of surgery, at least 2 hours prior to surgery, methylphenidate 10 mg and carvedilol 6.25 mg were administered. No other medications were taken within 12 hours of the procedure. The patient was considered an American Society of Anesthesiologists physical status score of 3 and Mallampati class I, based on her medical history and preanesthesia assessment. Prior to induction, vital parameters were blood pressure120/66 mm Hg, heart rate 80 beats per minute [bpm], and Spo_2_- 95% on room air. After an intravenous (IV) line was placed, midazolam 2 mg was given as a premedication, induction medications consisted of fentanyl 75 mg IV, lidocaine 100 mg IV, propofol 180 mg IV, and rocuronium 35 mg IV. Anesthesia was maintained with sevoflurane in air oxygen mixture at an average MAC value of 0.9. The procedure lasted for 2 hours. At the end of surgery, neuromuscular blockade was reversed with neostigmine and glycopyrrolate at 5 mg and 0.8 mg, respectively. Vital parameters remained stable intraoperatively, with a peak heart rate of 90 bpm, and maximum blood pressure of 132/70 mm Hg. Perioperative telemetry indicated a normal sinus rhythm. Postoperatively, vital parameters were not significantly different from preoperative measurements.

### Case 2

A 36-year-old female patient diagnosed with hypersomnia was scheduled to undergo hip arthroscopy for labral repair. The patient had a significant medical history of major depressive disorder, fibromyalgia, and allergic rhinitis. The patient had been taking methylphenidate extended release at a dose of 54 mg once daily for 1 year in association with short-acting methylphenidate, 10 mg BID. The physician suspected that her daytime sleepiness may be multifactorial and additional investigations ruled out obstructive sleep apnea, considering major depression and fibromyalgia as contributing factors.

Her medication also included lithium, 300 mg/d, valacyclovir HCl, cyclobenzaprine HCl, desvenlafaxine succinate, cetirizine, multiple vitamin, and mometasone.

A complete blood count, electrolyte profile, and electrocardiogram obtained prior surgery were within normal limits.

The patient adjusted the methylphenidate dose prior to surgery based on her hypersomnia symptoms, achieving a stable dose of 74 mg/d.

Twelve hours prior to surgery, the patient discontinued all her oral medication, including methylphenidate, based on the surgeon’s office standard procedure.

Physical examination on the day of surgery indicated BP - 113/70 mm Hg, pulse 66 bpm, oral temperature 98°F (36.7°C), and a respiration rate (RR) of 12 respirations per minute (rpm). The patient was classified as ASA class 2 and Mallampati class I based on her medical history and preanesthesia assessment. Standard monitoring indicated a blood pressure of 108/59 mm Hg and a heart rate of 78 bpm. After an IV line was established, midazolam was given (2 mg) as premedication. Anesthesia was carried out with fentanyl 100 mcg IV, lidocaine 60 mg IV, propofol 250 mg IV, and rocuronium 35 mg IV. Anesthesia was maintained with sevoflurane in air oxygen mixture at an average MAC value of 0.9. The procedure lasted for 3 hours. At the end of surgery, neuromuscular blockade was reversed with neostigmine and glycopyrrolate, 2 mg and 0.4 mg, respectively. Blood pressure, heart rate, and respiration rate remained stable throughout the surgery and postoperatively with a peak BP of 110/65 mm Hg, HR of 85 bpm, and RR of 16 rpm.

## Discussion

Our report is based on 2 different clinical experiences of patients on chronic methylphenidate use, undergoing anesthesia while taking versus withholding the drug treatment on the day of surgery.

Amphetamine drugs are commonly used for several medical conditions, including ADHD, narcolepsy, hypersomnia, and depression. Although the population of patients receiving psychostimulant therapy for these conditions is quite large, there is little available literature regarding the effects of these drugs on patients receiving general anesthesia. Historically, any psychostimulant medication was discontinued prior to surgery, because of concerns raised by early studies and anecdotal reports suggesting potential alteration of MAC requirements and serious cardiovascular complications related to amphetamine drugs effect on endogenous catecholamine release.^2,3^ There are situations, however, when urgent or emergent surgery might require the administration of general anesthesia on patients currently on analeptics use, within 24 hours of treatment. Methylphenidate has recently been shown to induce active emergence from isoflurane and propofol general anesthesia in rats.^5,6^ While the safety and efficacy of a similar approach in humans has yet to be studied, it is possible that amphetamine drugs could be used perioperatively to boost emergence and cognitive recovery following surgery. For these considerations, it will be important to understand the effects of chronic or acute amphetamine-like drugs treatment on patients undergoing general anesthesia in order to optimize anesthetic management.

Chiang^7^ considered dextroamphetamine a sympathomimetic amine that promotes the release of catecholamines (primarily dopamine and norepinephrine) from presynaptic nerve terminals. It also blocks the reuptake of catecholamines by competitive inhibition, with minor effect.^7^ Methylphenidate acts by inhibiting nerve terminal transporters that mediate the reuptake of dopamine and norepinephrine, affecting cerebral cortex and subcortical structures similarly to amphetamines. The drug is known to affect arousal-associated pathways controlled by the neurotransmitters dopamine, norepinephrine, and histamine.^8^

From the standpoint of anesthetic management and patient safety, the concerns for perioperative methylphenidate use are mainly related to cardiovascular stability and possible counteraction of sedatives and anesthetics. Methylphenidate and other amphetamine derivatives affect the release of endogenous catecholamines such as norepinephrine and dopamine, and thus act as indirect sympathomimetics. As such, they can exert effects on peripheral and central α- and β-adrenergic receptors affecting vascular tone, heart rate, and contractility. Studies assessing the cardiovascular effects of methylphenidate and dextroamphetamine in patients with ADHD have shown that acute use is associated with statistically, but not clinically, significant increases in heart rate and blood pressure.^9,10^ The cardiovascular responses of patients taking analeptics for hypersomnia, narcolepsy, or depression have not yet been evaluated.

It has been reported that chronic exposure to analeptics may cause catecholamine depletion or receptor downregulation.^2^ This may result in blunted catecholamine response to surgical stress. In the work originally performed on dogs by Johnston et al,^2^ animals chronically exposed to dextroamphetamine had decreased MAC requirements for halothane anesthesia as well as diminished hypertensive response to tyramine challenge.In a case report published in 1979, Samuels et al^3^ cited decreased MAC requirement leading to anesthetic overdose as a possible cause of cardiac arrest in a chronic amphetamine abuser undergoing cesarean section with halothane anesthesia.

Conversely, a study of 34 children taking methylphenidate and dexamphetamine for ADHD found no alteration of bispectral index or clinical depth of anesthesia at 1 MAC sevoflurane between patients continuing versus stopping the stimulant medication treatment on the day of surgery.^11^ In addition, case reports published in 2000 and 2006 demonstrated anesthesia without complication or alteration of MAC requirements in multiple patients on chronic amphetamine treatment who took the drug on the morning of surgery.^12,13^ The authors of these case reports recommended close cardiovascular monitoring and the use of direct-acting vasopressors such as epinephrine or phenylephrine for hypotension. Inadequate catecholamine response from indirect agents like ephedrine has been shown to occur in chronic amphetamine users.^14^

Both our case reports were focused on patients without ADHD, treated with stimulant medication for narcolepsy and hypersomnia.

Narcolepsy and hypersomnia are part of a group of disorders, known as the dyssomnias, which affect the quality, amount, or timing of sleep, because of abnormalities in the mechanisms generating sleep or the timing of sleep and wakefulness.^15^

No perioperative clinically significant variations in hemodynamic parameters were observed in our patients despite different therapeutic decisions to continue or to withhold methylphenidate on the day of surgery. We expected that the uninterrupted use of medication should have a beneficial effect concerning time of emergence from general anesthesia, postoperative oxygen saturation (Spo_2_), postoperative respiratory rate, apnea spells, and duration of stay in the postanesthesia care unit with minimal effect on perioperative hemodynamics. No clear guidelines are established for administration of anesthesia to patients with a diagnosis of dyssomnia and chronic stimulants use.

## Conclusion

The fact that the drug was continued throughout surgery, as was presented in our first case report, with no change in general anesthesia standard of care, suggests that methylphenidate may have a safe profile as a central nervous stimulant in patients undergoing general anesthesia. However, the use of a beta blocker in this particular patient with a history of hypertension, may have played an important stabilizing effect on heart rate.

Conclusions are complicated by the extent of the patients’ concurrent medications and comorbidities, the lack of significant changes in intraoperative blood pressure, heart rate, or sevoflurane requirement, despite different therapeutic decision, to continue versus to withhold methylphenidate treatment on the day of surgery. The surgical procedures were of limited duration - 2 hours for the removal of a vocal cord and 3 hours for the hip arthroscopic repair, suggesting a cautious interpretation of the results based on the length of the interventions as a confounding factor.Future prospective observational studies will help to better describe the effect of amphetamines on general anesthesia.
